# Identification of Potential Biomarkers in the Cervicovaginal Fluid by Metabolic Profiling for Preterm Birth

**DOI:** 10.3390/metabo10090349

**Published:** 2020-08-27

**Authors:** AbuZar Ansari, Heeyeon Lee, Young-Ah You, Youngae Jung, Sunwha Park, Soo Min Kim, Geum-Sook Hwang, Young Ju Kim

**Affiliations:** 1 Department of Obstetrics and Gynecology and Ewha Medical Research Institute, College of Medicine, Ewha Womans University, Seoul 07984, Korea; abu.zar.0313@outlook.com (A.A.); yerang02@naver.com (Y.-A.Y.); clarrissa@hanmail.net (S.P.); zeus_0218@naver.com (S.M.K.); 2 Integrated Metabolomics Research Group, Western Seoul Center, Korea Basic Science Institute, Seoul 03759, Korea; lhy322@kbsi.re.kr (H.L.); jya0819@kbsi.re.kr (Y.J.); 3 Department of Pharmacy, Ewha Womans University, Seoul 03760, Korea; 4 Department of Life Science, Ewha Womans University, Seoul 03760, Korea; 5 Department of Chemistry & Nano Science, Ewha Womans University, Seoul 03760, Korea

**Keywords:** cervicovaginal fluid, preterm birth, microbiota, metabolite, dysbiosis

## Abstract

During pregnancy, dysbiosis in the vaginal microbiota directly affects the metabolic profiles, which might impact preterm birth (PTB). In this study, we performed cervicovaginal fluid (CVF) metabolic profiling using nuclear magnetic resonance (NMR) spectroscopy and identified the metabolic markers for predicting PTB. In this nested case-control study, 43 South Korean pregnant women with PTB (n = 22), and term birth (TB; n = 21) were enrolled with their demographic profiles, and CVF samples were collected by vaginal swabs. The PTB group had two subgroups based on post-CVF sampling birth: PTB less than (PTB < 7 d) and more than 7 days (PTB ≥ 7 d). We observed significant differences in the gestational age at birth (GAB), cervical length (CL), and neonatal birth weight among the groups. The principal component analysis (PCA), and partial least square discriminant analysis (PLS-DA) scatter plot showed the separation between the PTB < 7 d group, and the TB group. Out of 28 identified metabolites, acetone, ethanol, ethylene glycol, formate, glycolate, isopropanol, methanol, and trimethylamine N-oxide (TMAO) were significantly increased in the PTB group compared with the TB group. The ROC curve analysis revealed that the acetone, ethylene glycol, formate, glycolate, isopropanol, methanol, and TMAO had the best predictive values for PTB. Additionally, the correlation analysis of these metabolites showed a strong negative correlation with GAB and CL. These metabolites could be beneficial markers for the clinical application of PTB prediction.

## 1. Introduction

Preterm birth (PTB), which is defined as the birth of a fetus in less than 37 weeks of the gestation period, is a serious problem of the neonate and maternal health with the risk of mortality and morbidity [1,2,3,4]. Approximately 15 million cases of PTB are registered annually, and this number is continuously rising all over the world [5]. Poor conditions of the medical, social, economic and particularly differences in ethnicity are prime factors of PTB [6]. Several measures have been introduced for the monitoring of PTB, like the examination of the previous history of PTB, a short cervix (< 25 mm), vaginal pH, pro-inflammatory cytokines (IL-6) in cervicovaginal fluid (CVF), or fetal fibronectin (FFN) analysis [7,8]. These measures merely offer superficial information, and are thus inadequate for the confirmation of the PTB-causing agent. In addition, reliability, accuracy, and data availability are also questionable for PTB prediction.

During the pregnancy, several physiological changes occur, particularly interaction between maternal microbiota and the fetus environment, which were found to be involved in health, and disease including PTB [6,9]. These interactions drive the hormonal and metabolic changes such as estrogen and progesterone levels, influencing the composition of vaginal microbiota communities [10]. In addition, maternal dietary intake during pregnancy, like a high carbohydrate or high protein diet, causes metabolic dysregulation and gut microbiota communities, leads to the dysbiosis of vaginal microbiota profile [11]. The dysbiosis of vaginal-microbiota occurs occasionally, which is considered the most common cause of ascending vaginal infection and other pregnancy diseases [12]. The shift from dominated *Lactobacillus* spp. to *Bacteroides* (Firmicutes), *Prevotella* (Bacteroidetes), *Klebsiella* (Proteobacteria), or *Mobiluncus* (Actinobacteria), spp. etc. are mainly associated with vaginal microbiota dysbiosis [6,13,14,15]. This dysbiosis directly impacts the production of pathogenic microbiota metabolites by changing the vaginal pH and inducing the release of pro-inflammatory cytokines (IL-6, IL-8, etc.), and immunological cells, that might be responsible for PTB [6,16,17,18]. Reportedly, previous studies showed that vaginal microbiota metabolites (high acetate, low succinate, etc.) are associated with PTB [19,20]. To date, very limited studies are available that have examined the putative microbiota metabolites for PTB. Furthermore, studies published have conflicting results that cannot be reliable for unearthing the specific predictive metabolite marker for PTB. Thus, to fulfill the present gap and for an in-depth understanding of vaginal microbiota metabolite inducing PTB, a concrete biomarker(s) is needed that can predict the underlying factors of PTB.

Presently, due to high sensitivity and accuracy, metabolomic analysis is profoundly used in the biomarker discovery area. Based on the metabolomic analysis, it is possible to predict the markers against a particular group of patients and create a metabolomic profile of the study group [21]. The vaginal microbiota metabolites profiling in CVF using an analytical approach such as the ^1^H-nuclear magnetic resonance (NMR) spectroscopy can reflect a specific signature for vaginal microbiota dysbiosis. Similarly, here, we hypothesized that determining the microbiota metabolite in the CVF samples by ^1^H-NMR may predict any reliably specific signatures for PTB that could be clinically useful for uncovering the vaginal environment and birth outcomes. This study aimed to characterize the CVF microbiota metabolites of women with PTB compared with term birth (TB) women and to predict the vaginal microbiota metabolite(s) association with PTB.

## 2. Results

### 2.1. Clinical Characteristics

**Figure 1:**
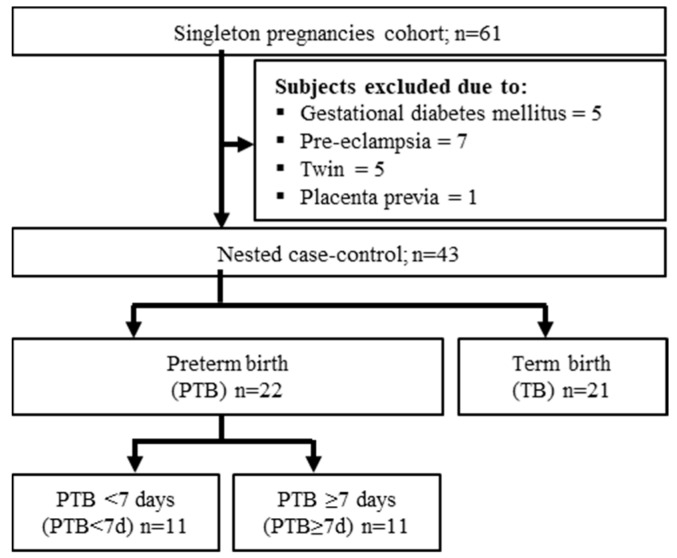
Study flowchart for the subject selection criteria. PTB: preterm birth; TB term birth; PTB < 7 d: preterm birth less than 7 days after cervicovaginal fluid (CVF) sampling, PTB ≥ 7 d: preterm birth more than 7 days after CVF sampling.

In this nested case-control study, 43 pregnant women were enrolled for the CVF metabolite analysis: among them, 22 had PTB and 21 had a TB outcome (Figure 1). The PTB had two subgroups: PTB less than 7 days (PTB < 7 d), and PTB more than 7 days (PTB ≥ 7 d) based on birth after CVF sampling. The clinical characteristics of the subjects were summarized in the table (Table 1). In the statistical analysis, there were significant differences in the gestational age at birth (GAB), cervical length (CL), neonatal birth weight, and APGAR (Appearance, Pulse, Grimace, Activity, Respiration) test between groups. No statistical difference was observed in the women’s age, the pre-body mass index (pre-BMI), gestational age at sampling (GAS), and blood parameters (WBC: white blood cell, and CRP: C-reactive protein) in the PTB group compared with the TB group (Table 1).

### 2.2. Metabolite Analysis of CVF Samples

The metabolites in CVF were analyzed using ^1^H-NMR spectroscopy coupled with multivariate data analysis to identify the metabolic characteristics of the PTB and TB groups. ^1^H NMR spectra obtained from the CVF samples are shown in the Supplementary Materials Figure S1. The spectral resonance of each metabolite was assigned based on the 800 MHz library in the Chenomx NMR suite (ver. 7.1, Edmonton, AB, Canada). Ambiguous peaks due to overlap or slight shifts were confirmed by spiking experiments with the commercial standard compounds. The 28 identified metabolites in the CVF samples were listed in the Supplementary Table S1. The identified CVF metabolites were as follows: 12 amino acids and conjugates (alanine, aspartate, creatine, glutamine, glycine, histidine, isoleucine, leucine, phenylalanine, threonine, tyrosine, valine), 5 alcohols and polyols (choline, ethanol, ethylene glycol, isopropanol, methanol), 1 ketone (acetone), 2 amines and polyamines (taurine, trimethylamine N-oxide (TMAO)), 1 carbohydrate (glucose), 1 nucleoside and conjugates (hypoxanthine) and 6 organic acids and derivatives (acetate, formate, glycolate, lactate, pyruvate, succinate).

### 2.3. Quantitative Analysis of the CVF Metabolite

**Figure 2:**
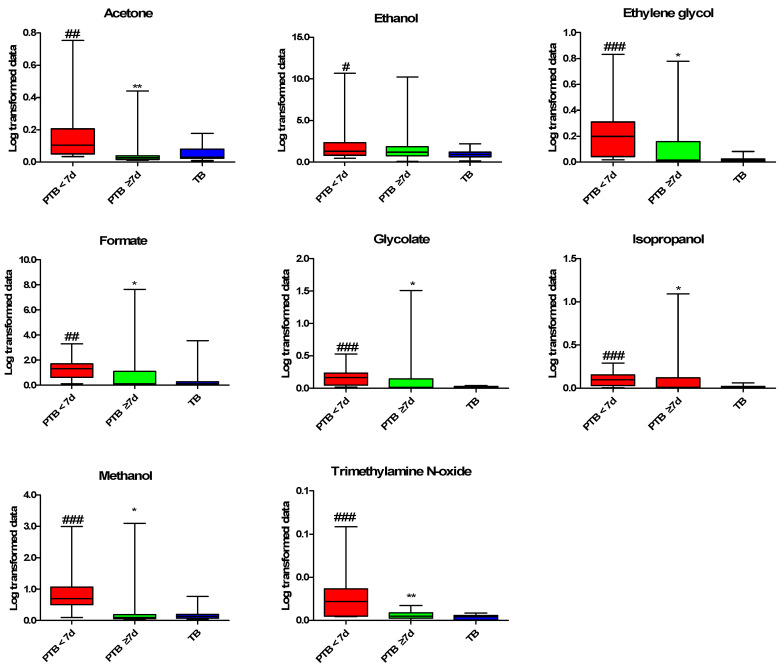
Increased metabolites profiling in CVF samples. Kruskal–Wallis test was used for statistical analysis. The PQN Log-transformed data were used for graphical representation. Data are presented as the mean ± standard deviation, and *p* < 0.05 considered as significant. #: PTB < 7 d vs. TB; *: PTB < 7 d vs. PTB ≥ 7 d. ^#^ *p* < 0.05, ^##^ *p* < 0.01, ^###^ *p* < 0.001, * *p* < 0.05, ** *p* < 0.01. PTB: preterm birth; PTB < 7 d: preterm birth less than 7 days after CVF sampling (n = 11); PTB ≥ 7 d: preterm birth more than 7 days after CVF sampling (n = 11); and TB: term birth (n = 21). PQN: Probabilistic quotient normalization.

**Figure 3:**
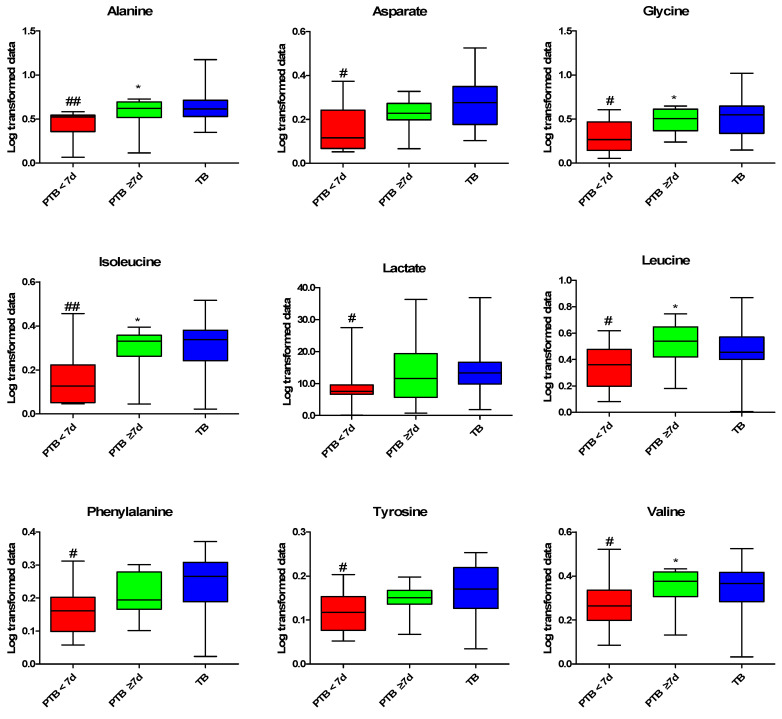
Decreased metabolites profiling in CVF. Kruskal–Wallis test was used for the statistical analysis. The PQN Log-transformed data were used for graphical representation Data are presented as the ± standard deviation, and *p* < 0.05 considered as significant. #: PTB < 7 d vs. TB, *: PTB < 7 d vs. PTB ≥ 7 d. ^#^ *p* < 0.05, ^##^ *p* < 0.01, * *p* < 005. PTB: preterm birth; PTB < 7 d: preterm birth less than 7 days after CVF sampling (n = 11); PTB > 7 d: preterm birth more than 7 days after CVF sampling (n = 11); and TB: term birth (n = 21). PQN: Probabilistic quotient normalization.

Differences between the PTB and TB groups were identified through multivariate analysis using the quantified CVF metabolites. In the principal component analysis (PCA) and partial least squares-discriminant analysis (PLS-DA) scatter plot derived from the quantified NMR data, the PTB < 7 d group was distinguished from the TB group by the first component, while the PTB ≥ 7 d and TB groups were not separated (Supplementary Materials Figure S2A,B). As shown in the variable importance plot (VIP) of the PLS-DA model between the PTB < 7 d and TB group, metabolites with a VIP score of >1 are as follows: glycolate, isopropanol, ethylene glycol, methanol, TMAO, ethanol, alanine, isoleucine, acetone, glycine, tyrosine, phenylalanine, aspartate and valine (Supplementary Materials Figure S2C). We performed statistical analysis to confirm the metabolites responsible for the differentiation between the PTB and TB groups. The eight metabolites were significantly increased in the PTB < 7 d group compared to the TB group, and the ethylene glycol, glycolate, methanol, isopropanol, and TMAO were the most significantly increased metabolites (*p* < 0.01) (Figure 2). By contrast, the nine metabolites significantly decreased in the PTB < 7 d group compared to the TB group, alanine, isoleucine were more significantly decreased (*p* < 0.01), than aspartate, glycine, lactate, leucine, phenylalanine, tyrosine, and valine (*p* < 0.05) (Figure 3).

### 2.4. Predictive Performance and Correlation Analysis for PTB

**Figure 4:**
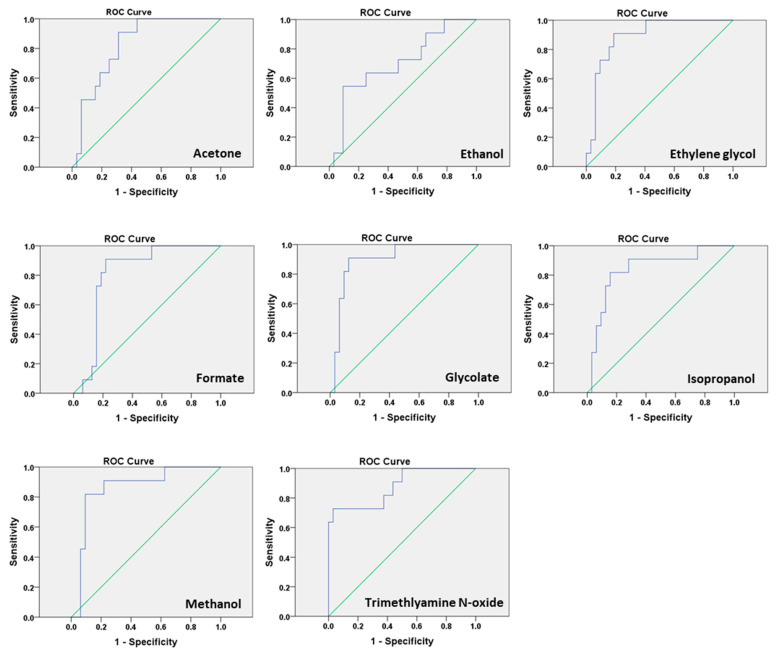
Receiver operating characteristics (ROC) curve analysis for metabolites: predictive performance of the biomarker for preterm birth using ROC curves of sensitivity and specificity.

Analysis of the area under the curve (AUC) of receiver operating characteristic (ROC) curves for the significant high metabolites showing the predictive value of more than 0.7 (Table 2, Figure 4) for PTB prediction. Out of 28 metabolites, 17 metabolites have *p* < 0.05 (cut off), while the calculated false discovery rate showed only 14 true significant metabolites (Supplementary Materials Table S2). We found that glycolate had the highest AUC (Table 2), thus we also calculated the combined model of the predictive performance of metabolites with and without CL (Supplementary Materials Table S3), and observed significantly high (*p* < 0.01) predictive values. The metabolites combined with clinical characteristics for the correlation by Pearson correlation analysis showed a significant negative correlation of GAB with CL (Table 3).

## 3. Discussion

This study characterized the CVF metabolite profiles in symptomatic and asymptomatic pregnant women at the mid-gestation period using ^1^H-NMR spectroscopy. This is the first study about the association of CVF metabolites and PTB in Korean pregnant women. Our results showed that glycolate, ethylene glycol, methanol, isopropanol, formate, acetone, ethanol, and TMAO levels were increased in PTB < 7 d after CVF sampling at the mid-gestation period. Identified metabolites mostly consisted of alcohol metabolites; acetone, ethanol, ethylene glycol, formate, glycolate, isopropanol, and methanol, except TMAO. Furthermore, these metabolites in CVF appear predictive of PTB in this subject. In addition, their increased levels were significantly correlated with CL, except with acetone and ethanol. Collectively, our results suggest that these metabolite analyses in CVF during pregnancy may be useful as predictive markers for PTB.

Vaginal microbiota generated metabolites that play an important role in gestational metabolism to maintain pregnancy [22]. Vaginal microbiota dysbiosis could affect microbiota-metabolites production, which might be the cause of PTB. High lactate reflects normal pregnancy which maintains vaginal pH and may help to protect vaginal infection, while a low level might cause adverse birth outcomes [6], as we observed among the low level of lactate in PTB < 7 d. Such a phenomenon among PTB subjects might be due to the decline of lactate-producing bacteria (*Lactobacillus* spp.) [6]. Acetate and succinate exhibit deleterious immunomodulatory function, and studies reveal that high acetate and low succinate in CVF associated with PTB [23,24], by disturbing the vaginal pH, induce pro-inflammatory markers (e.g., IL-1B, IL-6, TNF-a, and IL-8) through toll-like receptor (TLR) interaction [17,18,19,20]. Unfortunately, we did not observe the promising predictive value of acetate and succinate in PTB subjects in this cohort, and this result may be due to the presence of acetate/succinate producing microbiota (Bacteriodetes/Proteobacteria), and different ethnicity [19,20]. These studies reflect that the impact on the production of lactate or acetate/succinate might be the alteration of specific microbiota at the phyla/genus/species during the gestation period could induce PTB [13,25].

We observed a significantly high level of alcohol-fermented metabolites (ethylene glycol, glycolate, isopropanol, methanol, and formate). Identified high alcohol metabolites might be the presence of high alcohol-producing bacteria (Proteobacteria: *Klebsiella pneumonia*) [26], as we recently observed in the CVF of PTB women [14]. The high level of ethylene glycol in PTB < 7 might be due to the presence of the high colonization of Acinetobacter (biodegrade poly-ethylene glycol into ethylene glycol) or Candida spp. (biodegrade choline into ethylene glycol) [14,27,28,29]. In addition, the high TMAO in PTB < 7 d subjects, might be because of choline metabolism bacteria (Firmicutes), as we observed from the dominancy in CVF of PTB in our recent study [14]. Thus, the metabolites of PTB < 7 d after CVF sampling at mid-gestation may result from a response to a sudden change of vaginal microflora.

Ethanol, methanol, isopropanol, and ethylene glycol are major alcohol toxicants, which can regulate severe metabolic dysfunction [30]. Ethanol, methanol, or isopropanol are oxidized by bacterial alcohol dehydrogenase into the ethylene glycol/glycolate, formate, and acetone, respectively [30]. Methanol and isopropanol can cause the dysfunction of fetal development, and even death in the severe presence [31]. High formate is a diagnostic measure for methanol toxicity, while isopropanol toxicity screening is a clinical practice during pregnancy [32,33]. Ethanol derivatives i.e., ethylene, ethylene oxide, ethylene glycol, and glycolate are potent teratogens that may increase the risk of PTB, as we observed in our cohort a high level of ethylene glycol and glycolate in the PTB < 7 d group [34,35]. Ethanol appears to be inferior to betamimetics for preventing threatened preterm labor but we did not find an increased level of ethanol, it might be most ethanol converted into ethylene glycol and/or glycolate [36]. The consumption of polyethylene glycol or ethylene glycol though plastic-packed food items (juice, milk, etc.), during pregnancy might increase the availability of ethylene glycol as a threat to fetal development and birth outcome [37]. These toxicant metabolites are generated by bacterial alcohol dehydrogenase and interact with TLR which initiate inflammation by the upregulation of IL-6, and IL-8 concentrations and processed PTB [38,39,40]. Our results showed a high level of these alcohol metabolites in the PTB < 7 d group, with significant predictive values and highly significant correlation with GAB and the CL of these metabolites, associated with PTB.

TMAO is an osmolyte, and the concentration in the blood increases after consuming choline, or L-carnitine contained in the diet [41,42]. Choline or L-carnitine converted into TMA via microbial metabolism and finally into TMAO as a waster product is distributed in tissues and organs through hematogenic circulation [43]. Convincing evidence suggests an association between TMAO and inflammation with increased TNF-a, and IL-6 [44]. As we observed, the high level of TMAO in the PTB < 7 d group, with significant predictive value and correlated with GAB and CL, which might be the consumption of a choline or L-carnitine-containing diet during pregnancy [45,46,47].

These microbiota metabolites originated from dietary biomolecules (sugar, proteins, etc.) from the two basic phenomena, first through the bio-production of short-chain fatty acids (SCFAs; formate, acetate, etc.) or alcohol (ethylene glycol, isopropanol, ethanol, methanol, etc.) from fermenting sugar (maltose, sucrose, fructose, glucose) and second, derived through a bioconversion process like TMAO from choline in the presence of microbiota [48,49] (Graphical abstract). In this cohort, we identified significantly high metabolites belonging to an alcohol fermentation metabolism (ethanol, methanol, isopropanol, ethylene glycol, and formate), except TMAO. The high availability of these metabolites attains statistical significance in the PTB > 7 d group, which might be due to the consumption of a high amount of fermented sugar and/or protein during pregnancy, influencing the birth gestational age and resulting in PTB with a low birth weight infant [50,51,52].

We acknowledge several limitations in this nested case-control study. The microbiota analysis, the small number of subjects and homogeneity in the ethnicity of the subjects were the primary limitations. The vaginal pH and FFN are the most utilized clinical assessment marker for distinguishing PTB women from TB women, but we did not detect the pH and FFN level, which was another limitations. In addition, we did not collect the information about maternal dietary nutrients (sugar, protein, or alcohol, etc.), the frequency of plastic-packed food consumption during pregnancy, and drugs or medications.

In conclusion, we identified eight microbiota metabolites; acetone, ethanol, ethylene glycol, formate, glycolate, isopropanol, methanol, and TMAO with a significantly high level, with the best predictive values except ethanol. These results revealed that these metabolites could be potential candidates for PTB markers in PTB management. Additionally, more emphasis and experiments are needed with a higher subject number for further confirming these predictive markers for the PTB clinical applications.

## 4. Materials and Methods

### 4.1. Study Subjects

The study subjects were enrolled during the period 2018–2019, at Ewha Womans University, Mokdong Hospital, Republic of Korea (Ethical Research Committee approval no. EUMC 2018-07-007). The subjects of the study were outpatients with asymptomatic pregnant women or pregnant women hospitalized with symptoms of preterm labor (PTL) and/or preterm premature rupture of membranes (pPROM) from 15 to 35 weeks. After the exclusion of subjects who were diagnosed with gestational diabetes mellitus, preeclampsia, hemolysis, elevated liver enzymes, a low platelet count (HELLP) syndrome, incompetent internal Os of the cervix (IIOC), and placenta previa, a total of 43 women with singleton pregnancies were included in this nested case-control study (Figure 1). All the included women were divided into two main groups: preterm birth (PTB, n = 22) with the gestation age of 32.2 ± 3.9 weeks and term birth (TB; n = 21) with the gestation age of 32.6 ± 3.8 weeks. The PTB group was further divided into two subgroups based on birth after the CVF sampling: birth less than 7 days (PTB < 7 d; n = 11) after CVF sampling, and birth more than 7 days (PTB ≥ 7 d; n = 11) after CVF sampling (Figure 1). We collected the pregnancy outcome through the subject chart review. The CVF sample was obtained from the posterior fornix of the vagina from the pregnant women with PTB and TB from vaginal swabs by PAP BRUSH (Bion Life Science Co. Ltd., Gyeonggi-do, Korea), followed by dispensing into the sterile phosphate buffer saline (PBS) tube, labeling and storage (at −80 °C) until metabolomic profiling. The CVF sample was collected before any vaginal examination or clinical treatment intervention such as therapy with antibiotics, steroids, and tocolytics.

### 4.2. CVF Sample Preparation for NMR Analysis

CVF samples were stored at −80 °C until NMR analysis. Before the NMR experiment, the frozen CVF samples were thawed at room temperature and vortexed. The 200 µL of CVF samples were mixed with 450 µL of 0.2 M sodium phosphate buffer (pH 7.00) in D2O. After adjusting the pH to 7.00 ± 0.05, the samples were centrifuged at 17,000 rpm for 20 min at 4 °C and 600 µL aliquots of supernatant were transferred into 5 mm NMR tubes for analysis.

### 4.3. H-NMR Experiment

^1^H-NMR spectra of the CVF samples were acquired on a Bruker Avance III HD 800 MHz FT-NMR spectrometer using a 5 mm triple-resonance inverse (TCI) cryoprobe with Z-Gradients (Bruker BioSpin Co., Billerica, MA, USA). To acquire one-dimensional (1D) 1H spectra of the CVF samples, Bruker standard 1D 1H T2 filter (Car–Purcell–Meiboom–Gill (CPMG)) pulse sequence was used with the relaxation delay (RD) = 2.0 s, CPMG echo delay (τ) = 0.2 ms, repetitions number (n) = 256, dummy scans = 16, loops = 160, and acquisition time (Acq) = 2.0 s. The water signal was suppressed at the water peak during RD. Free induction decay (FID) was acquired with a spectral width of 20 ppm for 64,000 data points.

As the reference sample, an ERETIC (electronic reference to access in vivo concentrations) reference (ER) sample was used [53]. In the ER sample, valine was included as a reference molecule. The reason why valine was used as a reference molecule is that the methyl group of valine does not interact with serum macromolecules such as human serum albumin (HSA) and fatted HSA (fHSA). Thus, the ER sample was prepared by mixing 150 μL of CVF, 12 μL of 100 mM valine, and 438 μL of D2O, to achieve a final valine concentration of 2 mM.

### 4.4. Data Processing of the ^1^H-NMR Spectra and Multivariate Analysis

All ^1^H-NMR spectra were phased and calibrated using TopSpin software (ver. 3.1, (Bruker BioSpin Co., Billerica, MA, USA). The CVF spectra were calibrated using the chemical shift of formate at 8.445 ppm. The synthetic ERETIC signal was used instead of trimethylsilylpropionic acid (TSP) in the CVF sample. The internal NMR reference chemicals, DSS or TSP, are known to interact with serum macromolecules unless serum has been deproteinized. For this reason, DSS or TSP cannot be used as reference chemicals in serum samples. Since we confirmed that a small amount of blood was present in the CVF sample during the sample preparation, we used the ERETIC peak as a reference peak [54,55]. The ERETIC peak of the CVF samples and the intensities of the identified metabolites were used to determine their relative concentrations. The ERETIC peak parameters were defined as follows: ERETIC peak position = 0.0 ppm, line width = 0.4 Hz, integral = 2.2 × 10^8^ and correction factor = 1.

The processed NMR spectra were imported into Chenomx (Version 7.1, Edmonton, AB, Canada) for the identification and quantification of metabolites. The 800 MHz Chenomx library was used to identify and quantify the individual compounds. To avoid inaccurate integral normalization due to significant changes in massive amounts of single metabolites in samples [56], the quantification results were normalized by Probabilistic Quotient (PQ) normalization. Ambiguous peaks due to overlap or slight shifts were confirmed by spiking experiments.

The quantified data were then imported into SIMCA-P+ version 12.0 (Umetrics, Umea, Sweden), and data were unit-variance (UV) scaled for multivariate statistical analysis. Principal components analysis (PCA) was performed to obtain the variation among the groups, and partial least squares discriminant analysis (PLS-DA) was used as a classification method. The qualities of the models were described using R2 and Q2 values. R2 is defined as the proportion of variance in the data explained by the model. Q2 is defined as the proportion of variance in the data predictable by the model.

### 4.5. Statistical Analysis

The clinical characteristics and analyzed metabolites were compared using the Kruskal–Wallis test. The false discovery rate was calculated to find truly significant results using the metabolites *p*-value. Pearson’s correlation was used to obtain the relationship between the clinical (Pre-BMI, GAB, CL, WBC, and CRP), and increased metabolites’ profiles. The receiver operating characteristic (ROC) curves analysis of significantly up-regulated metabolites of PTB and TB were used to generate the area under the curve (AUC) plot. *p* < 0.05 was considered as statistically significant. Statistical Package for Social Sciences (SPSS, Version 2.0 Chicago, IL, USA), and online MEDCALC software were used for the statistical analysis.

## Figures and Tables

**Table 1:** Clinical characteristics of subjects.

Individuals	Characteristics	PTB < 7 Days (n = 11)	PTB ≥ 7 Days (n = 11)	TB (n = 21)
Mother	Age (years)	33.3 ± 3	30.8 ± 5	33.5 ± 4
	Pre-pregnancy-BMI (kg/m^3^)	21.5 ± 2.7	20.4 ± 3	21.5 ± 2.3
	GAS (weeks)	26.3 ± 5.2	25.4 ± 5.4	23.5 ± 3.4
	GAB (weeks)	26.6 ± 5.2 ^a,b^	33.4 ± 3 ^a,c^	39.3 ± 0.8 ^b,c^
	CL (mm)	9.5 ± 13.8 ^a,b^	16.5 ± 13.5 ^a,c^	29.7 ± 4.9 ^b,c^
	WBC (1 × 10^3^/µL)	11.5 ± 4.2	10.6 ± 2.6	9.3 ± 1.7
	CRP (mg/dL)	1 ± 1.7	0.4 ± 0.4	0.3 ± 0.2
Infant	Birth weight (g)	1089.2 ± 596.8 ^a,b^	2078.6 ± 672.3 ^a,c^	3282.4 ± 221.2 ^b,c^
	APGAR 1 (min)	5 ± 2.8 ^b^	6.6 ± 2.8 ^c^	9.8 ± 0.6 ^b,c^
	APGAR 5 (min)	7.1 ± 1.9 ^a,b^	8.7 ± 1.3 ^a,c^	10 ± 0.2 ^b,c^

Kruskal–Wallis test was used for the statistical analysis. Data are presented as the mean ± standard deviation, and *p* < 0.05 considered as significant. ^a^: PTB < 7 d vs. PTB ≥ 7 d; ^b^: PTB < 7 d vs. TB; ^c^: PTB ≥ 7 d vs. TB. PTB, preterm birth, TB, term birth; BMI: body mass index; GAS: gestational age at sampling; GAB: gestation age at birth; CL: cervical length; WBC: white blood cell; CRP: C-reactive protein; APGAR: appearance, pulse, grimace, activity, respiration.

**Table 2:** Predictive performance of significant high metabolites in CVF.

Metabolites	AUC.	SEN.	SPE.	PPV.	NPV.	95% CI.	*p*-Value
Acetone	0.82	90.91%	68.75%	50.00%	95.65%	0.70–0.95	0.0015
Ethanol	0.71	54.55%	90.62%	66.67%	85.29%	0.52–0.89	0.0481
Ethylene glycol	0.89	90.91%	81.25%	62.50%	96.30%	0.79–0.99	0.0001
Formate	0.81	90.91%	78.12%	58.82%	96.15%	0.68–0.95	0.0022
Glycolate	0.90	90.91%	87.50%	71.43%	96.55%	0.80–1.00	0.0001
Isopropanol	0.84	81.82%	84.38%	64.29%	93.10%	0.70–0.98	0.0008
Methanol	0.86	81.82%	90.62%	75.00%	93.55%	0.73–0.99	0.0004
Trimethylamine N-oxide	0.88	72.73%	96.88%	88.89%	91.18%	0.75–1.00	0.0002

Receiver operating characteristics (ROC) curve analysis was performed for statistical analysis, and *p* < 0.05 considered as significant. AUC: area under the curve; SEN: sensitivity; SPE: specificity; PPV: positive predictive value; NPV: negative predictive value; CI: confidence interval.

**Table 3:** Correlation analysis between the metabolites and clinical parameters.

Metabolites	Pre-BMI	GAB	CL	WBC	CRP
Ethylene glycol	−0.208	−0.584 ***	−0.505 ***	0.134	0.187
Formate	−0.030	−0.243	−0.316 *	−0.021	0.103
Glycolate	−0.290	−0.345 *	−0.305 *	0.226	0.181
Isopropanol	−0.048	−0.283	−0.302 *	−0.095	0.059
Methanol	−0.125	−0.352 *	−0.337 *	−0.021	0.093
Trimethylamine N-oxide	−0.062	−0.682 ***	−0.400 **	0.044	0.167

Pearson correlation analysis was performed for the statistical analysis. BMI: body mass index; GAB: gestation age at birth; CL: cervical length; WBC: white blood cell; CRP: C-reactive protein. * *p* < 0.05; ** *p* < 0.01; *** *p* < 0.001.

## Supplementary Materials

The following are available online at https://www.mdpi.com/2218-1989/10/9/349/s1, Figure S1: Representative 800 MHz ^1^H-NMR spectrum obtained from the CVF sample, Figure S2: Principal component analysis, Table S1: ^1^H chemical shift and relative concentrations of the identified metabolites in CVF from PTB and TB, Table S2: The false discovery rate (FDR) of metabolites, Table S3: Predictive performance for the combined model of metabolites and CL.
