# Supplementary material for: Identification of Potential Biomarkers in the Cervicovaginal Fluid by Metabolic Profiling for Preterm Birth
Source: Metabolites. 2020 Aug 27;10(9):349. doi: 10.3390/metabo10090349 (PMC7570126; doi:10.3390/metabo10090349)
Supplement: Supplementary file 1 [file metabolites-10-00349-s001.pdf]

Original Article

Supplementary Materials: Identification of potential biomarkers in the cervicovaginal fluid by metabolic profiling for preterm birth

AbuZar Ansari, Heeyeon Lee, Young-Ah You, Youngae Jung, Sunwha Park, Soo Min Kim, Geum-Sook Hwang, Young Ju Kim

Supplementary Table 1. ^{1}H-NMR chemical shift and relative concentrations of identified metabolites in CVF from PTB and TB.

Metabolites	Chemical shifts (δ ^{1}H ppm)
Acetate	1.90 (s)
Acetone	2.22 (s)
Alanine	1.47 (d), 3.78(q)
Aspartate	2.67 (dd), 2.80 (dd), 3.89 (dd)
Choline	3.19 (s)
Creatine	3.02 (s), 3.92 (s)
Ethanol	1.17 (t), 3.65 (q)
Ethylene glycol	3.66 (s)
Formate	8.44 (s)
Glucose	3.23 (t), 3.40-3.90 (m), 4.64 (d), 5.23 (d)
Glutamate	2.05 (m), 2.12 (m), 2.30-2.38 (m), 3.75 (dd)
Glycine	3.55 (s)
Glycolate	3.93 (s)
Histidine	7.11 (s), 7.94 (s)
Hypoxanthine	8.18 (s), 8.20 (s)
Isoleucine	0.93 (t), 1.00 (d)
Isopropanol	1.16 (d), 4.02 (m)
Lactate	1.32 (d), 4.10 (q)
Leucine	0.95 (dd), 1.65-1.75 (m)
Methanol	3.34 (s)
Phenylalanine	7.32 (d), 7.37 (t), 7.42 (t)
Pyruvate	2.36 (s)
Succinate	2.39 (s)
Taurine	3.25 (t), 3.41 (t)
Threonine	4.24 (m)
Trimethylamine N-oxide	3.25 (s)
Tyrosine	6.89 (d), 7.18 (d)
Valine	0.98 (d), 1.03 (d), 2.26 (m), 3.60 (d)

Letters in parentheses denote the peak multiplicities: s, singlet; d, double; t, triplet; dd, doublet of doublet and m, multiplets.

Supplementary Table 2: The false discovery rate (FDR) of metabolites.

Rank	Metabolite	p-Value	q-value
1	Glycolate	**0.0001**	**0.0025**
2	Ethylene glycol	**0.0001**	**0.0017**
3	Trimethylamine N-oxide	**0.0002**	**0.0020**
4	Methanol	**0.0004**	**0.0029**
5	Isopropanol	**0.0008**	**0.0047**
6	Acetone	**0.0015**	**0.0070**
7	Formate	**0.0022**	**0.0088**
8	Alanine	**0.0029**	**0.0101**
9	Isoleucine	**0.0041**	**0.0129**
10	Glycine	**0.0064**	**0.0179**
11	Aspartate	**0.0104**	**0.0266**
12	Leucine	**0.0113**	**0.0264**
13	Phenylalanine	**0.0155**	**0.0333**
14	Valine	**0.0242**	**0.0483**
15	Tyrosine	**0.0279**	0.0520
16	Lactate	**0.0299**	0.0524
17	Ethanol	**0.0481**	0.0793
18	Glutamate	0.0895	0.1393
19	Choline	0.0949	0.1399
20	Pyruvate	0.0949	0.1329
21	Acetate	0.1191	0.1587
22	Histidine	0.2902	0.3693
23	Hypoxanthine	0.3163	0.3851
24	Succinate	0.3731	0.4353
25	Threonine	0.5220	0.5847
26	Glucose	0.6163	0.6638
27	Creatine	0.7175	0.7440
28	Taurine	0.9778	0.9778

Supplementary Table 3 Predictive performance for the combined model of metabolites and CL.

Combined Model	AUC.	SEN.	SPE.	PPV.	NPV.	95% CI.	*p*-Value
Glycolate + Isopropanol	0.88	90.9	84.4	66.7	96.4	0.74 – 0.96	<0.0001
Glycolate + Methanol	0.88	90.9	87.5	71.4	96.5	0.75 – 0.96	<0.0001
Glycolate + Formate	0.84	90.9	78.1	58.8	96.1	0.70 – 0.93	<0.0001
Glcolat + Isopropanol + Methanol	0.77	72.7	90.6	72.7	90.6	0.62 - 0.89	0.0050
Glycolate + Isopraponol + Formate	0.83	90.9	78.1	58.8	96.1	0.68 – 0.93	<0.0001
Glycolate + Isopropanol + CL	0.81	90.9	75.0	55.6	96.0	0.66 – 0.91	0.0010
Glycolate + Methanol + CL	0.80	90.9	75.0	55.6	96.0	0.65 – 0.91	0.0010
Glycolate + Formate + CL	0.82	90.9	75.0	55.6	96.0	0.68 – 0.92	0.0010
Glcolat + Isopropanol + Methanol + CL	0.81	90.9	75.0	55.6	96.0	0.66 – 0.91	0.0010
Glycolate + Isopraponol + Formate + CL	0.81	81.8	81.3	60.0	92.9	0.66 – 0.92	0.0010

Receiver Operating Characteristics (ROC) curve analysis was performed for statistical analysis, and $p<0.05$ considered as significant. AUC: Area under the curve; SEN: Sensitivity; SPE: Specificity; PPV: Positive predictive value; NPV: Negative predictive value; CI: Confidence interval; CL: Cervical length.

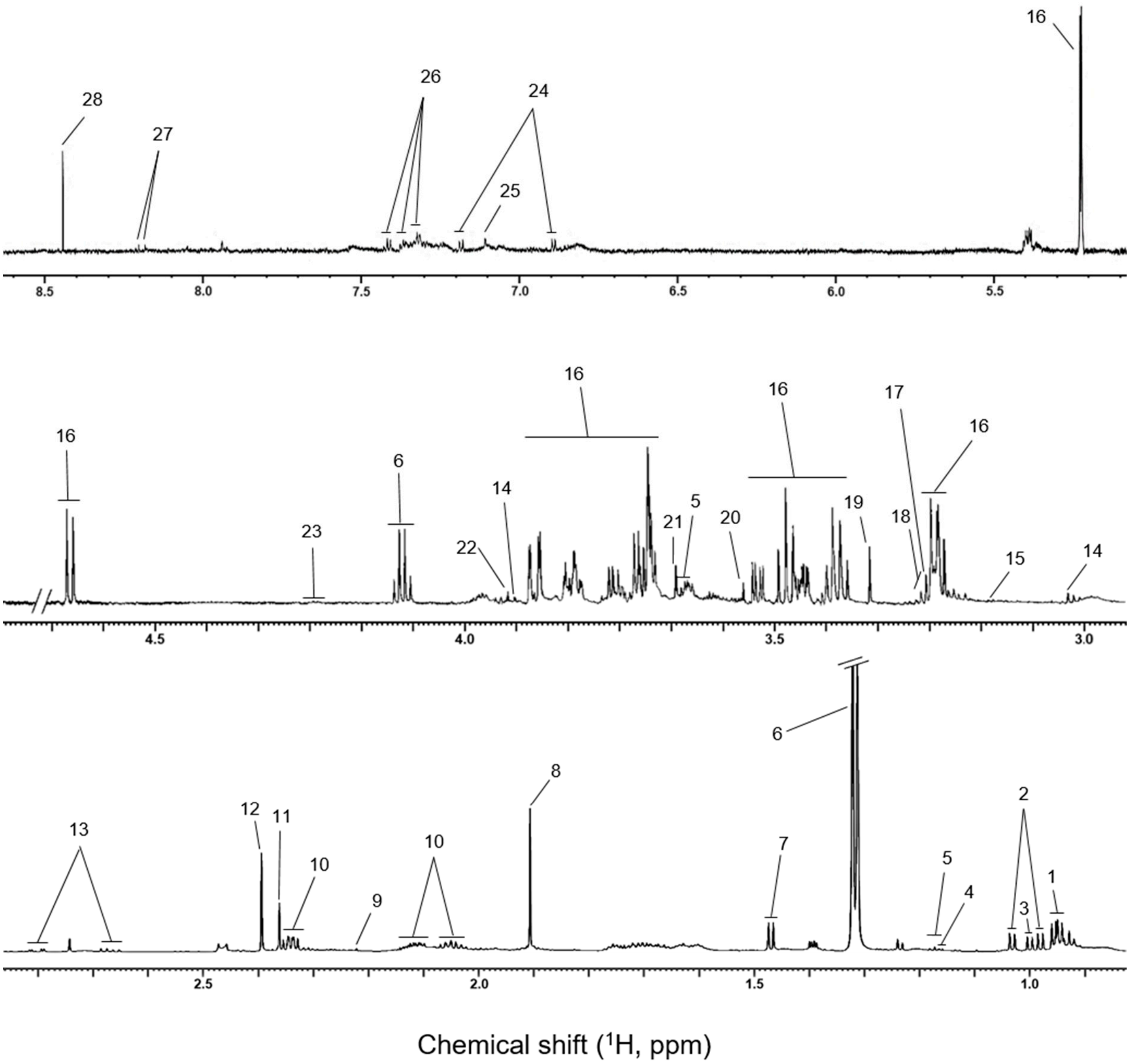

Supplementary Figure 1. Representative 800-MHz ^{1}H-NMR spectrum obtained from the CVF sample. Key: 1. leucine; 2. valine; 3. isoleucine; 4. isopropanol; 5. ethanol; 6. lactate; 7. alanine; 8. acetate; 9. acetone; 10. glutamate; 11. pyruvate; 12. succinate; 13. aspartate; 14. creatine; 15. choline; 16. glucose; 17. trimethylamine N-oxide; 18. taurine; 19. methanol; 20. glycine; 21. ethylene glycol; 22. glycolate; 23. threonine; 24. tyrosine; 25. histidine; 26. phenylalanine; 27. hypoxanthine; 28. formate.

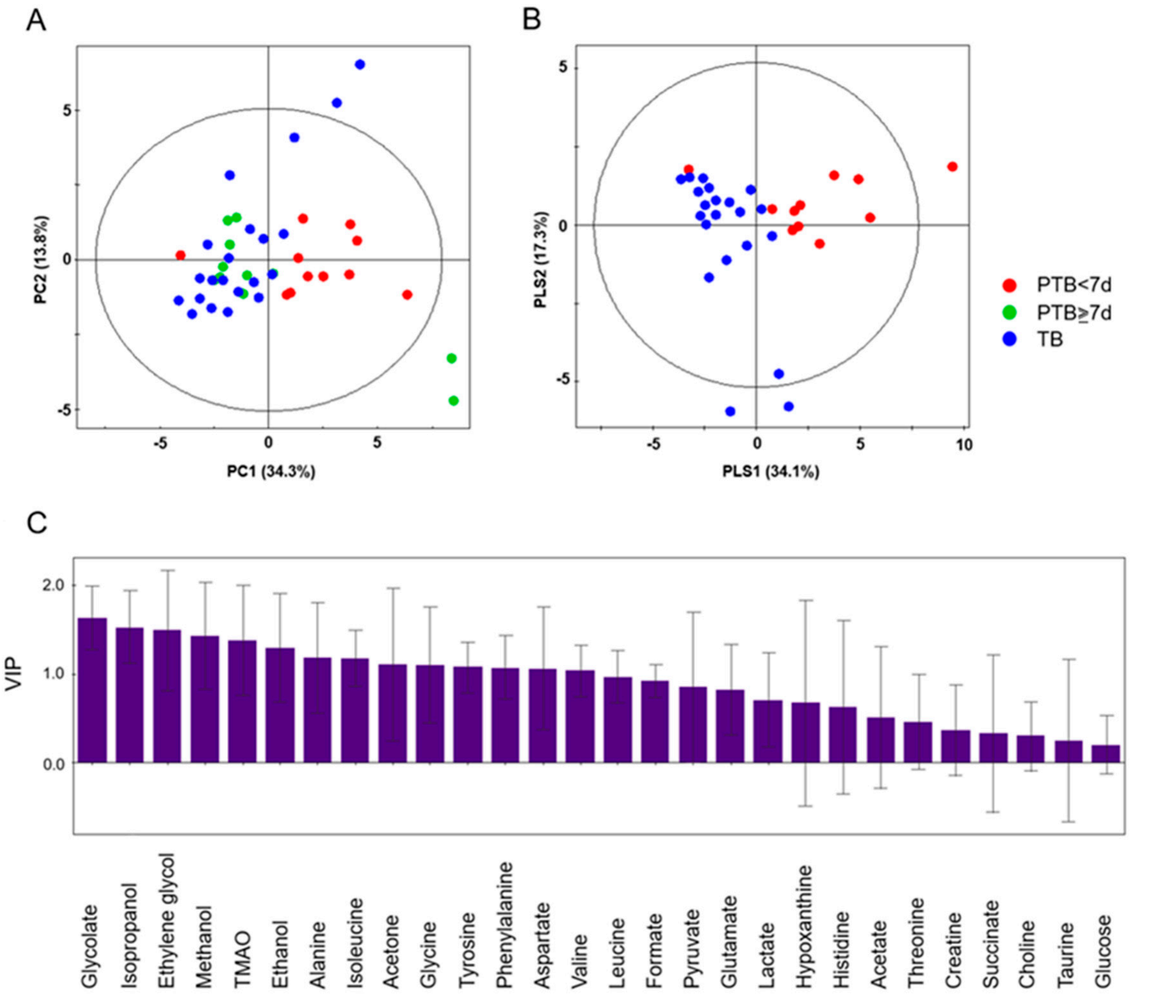

Supplementary Figure 2. Multivariate statistical analyses of CVF samples. (A) The Principal Component Analysis (PCA) and (B) Partial Least Squares-Discriminant Analysis (PLS-DA) scatter plot obtained from the quantified NMR data (PCA: R^2X=0.482, Q^2=0.186; PLS-DA: R^2X=0.515, $R^2Y=0.561$, Q^2=0.322). (C) Variable importance plot (VIP) of the PLS-DA model. PTB>7d: Preterm birth less than 7 days after CVF sampling; PTB<7d: Preterm birth more than 7 days after CVF sampling; and TB: Term birth.
